# Evaluation of the effectiveness of the Family Nurse Partnership home visiting programme in first time young mothers in Scotland: a protocol for a natural experiment

**DOI:** 10.23889/ijpds.v5i1.1154

**Published:** 2020-03-16

**Authors:** F Lugg-Widger, M Robling, M Lau, S Paranjothy, J Pell, J Sanders, J White, R Cannings-John

**Affiliations:** 1 Centre for Trials Research, Cardiff University, Cardiff, CF14 4YS.; 2 Division of Population Medicine, Cardiff University, Cardiff, CF14 4YS.; 3 Institute for Health and Wellbeing, University of Glasgow, Glasgow, G12 8RZ.; 4 School of Healthcare Sciences, Cardiff University, Cardiff, CF14 4XN.; 5 Centre for the Development and Evaluation of Complex Interventions for Public Health Improvement (DECIPHer), Cardiff University, Cardiff, CF10 3BD.

## Abstract

**Introduction:**

Individual, social and economic circumstances faced by young mothers (19 years or under) can challenge a successful start in life for their children. Intervening early might enhance life chances for both mother and child. The Family Nurse Partnership (FNP) is an intensive nurse-led home visiting programme developed in the US which aims to improve prenatal health behaviours, birth outcomes, child development and health outcomes, and maternal life course. Establishing evidence of effectiveness beyond the original US setting is important to understand where further adaptation is required within a country specific context.

**Objective:**

This study will form one strand of the Scottish Government’s plan to evaluate the effectiveness of FNP as compared to usual care for mothers and their children in Scotland and will focus only on outcomes that can be identified using routine administrative data systems.

**Methods:**

This study is a natural experiment with a case-cohort design using linked anonymised routine health, educational and social care data. Cases will be women enrolled as FNP Clients in ten NHS Health Boards in Scotland and Controls will be women who met FNP eligibility criteria but were pregnant at a time when the programme was not recruiting. Outcomes are mapped to the Scottish FNP logic model. All comparative analyses will be pre-specified, conducted on an intention to treat basis and will use multilevel regression models to compare outcomes between groups.

**Discussion:**

The study protocol is based upon the specification of FNP commissioned by the Scottish Government. This study design is novel for the evaluation of the FNP/NFP programmes which are primarily evaluated with an RCT. Outcomes included within the study have been selected on the basis that they are outcomes FNP aims to influence and where there is routine data available to assess the outcome

## Introduction

### The importance of early life experiences for child health and development

Individual, social and economic circumstances faced by young mothers (19 years or under) can challenge a successful start in life for their children. Children born to young mothers have lower birth weights, are less likely to receive breast milk, have higher mortality rates, are more likely to suffer accidents, experience lower educational attainment, more emotional and behavioural problems, and become young parents themselves [1–4]. Young and economically disadvantaged mothers are also more likely to smoke thus increasing the risk of adverse health outcomes for themselves and their children [5]. 

### Intervening to support families

Intervening early in the lives of families with young mothers might enhance life chances for both mother and child. The Nurse Family Partnership (NFP) is an intensive preventative home-visiting service which involves up to 64 structured home visits across pregnancy, infancy and toddlerhood by specially recruited and trained family nurses from early pregnancy until children are 2 years of age. It is one of 12 programmes meeting Home Visiting Evidence of Effectiveness (HomVEE) criteria for an evidence based intervention [6]. Developed in the US with young first-time mothers, the programme aims to address the problems of poor birth outcomes, child abuse and neglect, and the diminished economic self-sufficiency of mothers [7]. The NFP draws upon theories of human ecology, self-efficacy and human attachment [8–10]. In three US trials, the NFP has demonstrated improvements in prenatal health behaviours and birth outcomes, improvements in sensitive care giving, reductions in child injuries, abuse and neglect, improvements in maternal life course (e.g. greater workforce participation, fewer subsequent pregnancies, and reduction in welfare requirements) and improvements in child and adolescent functioning [9, 11–18].

### International replication of NFP

A phased process of programme replication (adaptation to local context, pilot testing for feasibility and acceptability, randomised controlled trial (RCT), replication and expansion) underpins the adoption of the NFP in non-US settings. In England, the Family Nurse Partnership (FNP) programme was introduced in April 2007 across 10 pilot sites and was evaluated in the Building Blocks RCT (BB:0-2) at 18 sites which reported in October 2015 [19, 20]. The primary outcomes measured (as selected on the basis of three US trials and government priorities) were smoking in late pregnancy, birthweight, child admissions to hospital including A&E attendance before their second birthday and subsequent pregnancies within 24 months of the mother’s first child being born. The trial also looked at a range of secondary outcomes, including child development up to age two. The researchers found that adding FNP to the health and social care provided in England had no additional benefit on the primary outcomes while between-group differences were found for some secondary outcomes (including intention-to-breastfeed, maternally reported child cognitive development, language development). In a Dutch trial of NFP (the VoorZorg trial) where teenage pregnancy rates are much lower than in the UK, the intervention was offered following a two-stage selection process identifying women with multiple risk factors (e.g. psychological, health, economic, social). VoorZorg found NFP lowered rates of smoking in late pregnancy, and increased rates of breast feeding at six months post-birth [21, 22]. 

### FNP in Scotland

FNP was first introduced to Edinburgh Community Health Partnership (CHP) NHS Lothian in 2010 and was subject to an independent evaluation in 2013 focussing on its implementation in a Scottish context [23]. The evaluation concluded that it was possible to implement FNP with fidelity in Scotland and could plausibly deliver improved outcomes in pregnancy, child health and development, maternal health, and self-efficacy. The Scottish Government aimed to build on and supplement the existing national and international evidence base for FNP, and assess effectiveness and opportunities for optimisation of the programme, to guide decision making on maternal and child health in Scotland. Part of the licencing agreement for FNP stipulates that an RCT should be undertaken. In 2015, an independent evaluability assessment for FNP in Scotland was undertaken to identify options for carrying out an RCT style evaluation. The outcome of the evaluability assessment was that a natural experimental study using retrospective routinely collected data be undertaken as the preferred method for evaluating impact [24].

## Methods

### Study aim

This study will use anonymised individual level routinely collected data to evaluate the effectiveness of FNP in Scotland. The aim of this study is to examine the association between the provision of FNP, when added to existing services, and a range of outcomes covering maternal and child health, child development, and parental life course compared to existing services alone for first time teenage mothers.

### Study design

Following the Medical Research Council’s (MRC) guidance on natural experiments, the FNP Programme in Scotland will be evaluated using a case-cohort design [25]. A linked anonymised research database will be generated to compare routinely available health, education, and social care data between mothers to be in receipt of FNP (Cases) and a Control sample who would meet the criteria for FNP but not in receipt of support (Controls). 

### Study population

The population will be all women eligible for the FNP Programme from 1st January 2009 to 31st March 2016 and their first-born child(ren). 

### Identifying Cases and Controls

#### Cases

Cases will be women and first-borns enrolled as FNP Clients in the ten participating Scottish Health Boards (HB) since its initiation from 1st January 2010 to 31st March 2016. Eligible women (criteria in Table 1A) are usually referred to the local FNP team after their antenatal booking appointment at around 12 weeks gestation and are required to be enrolled in the programme before 29 weeks. Cases will be identified from the FNP Scottish Information System (FNP SIS).

**Table 1: Maternal baseline characteristics measured at (or before) date of antenatal booking/enrolment:** ^1^Either Health Board level or smaller geographical area such as Community Health Partnership level or where a recruitment area was defined by travel time ^2^Derived by Electronic Data Research and Innovation Service (eDRIS) SMR: Scottish Morbidity Record

A. FNP Clients eligibility criteria	B. Eligibility criteria applied to fields within SMR 02 dataset to identify Controls
1. Living in an FNP-recruiting NHS Health Board	Postcode at antenatal booking mapped onto each FNP recruiting area1
2. First time mothers-to-be (women are eligible if a previous pregnancy resulted in a miscarriage, stillbirth or termination)	No previous live birth
3. Aged 19 years or younger at time of last menstrual period (LMP)	Derived variable2 based on date of birth and date of LMP
4. Less than 28+6 weeks gestation at enrolment into FNP	Gestation at booking date (derived variable2 based on date of booking and date of LMP)

Exclusion criteria

5. Mother-to-be will relinquish baby at birth	Not measurable at recruitment – minimal risk to numbers
6. Moving outside of the FNP catchment area before programme end	Not measurable at recruitment – minimal risk

#### Controls

The FNP eligibility criteria in Table 1A will be applied to fields in the Scottish Morbidity Record (SMR) 02 Maternity Inpatients and Day Cases dataset to identify potential Controls for each of the areas within the ten participating HBs. Controls’ antenatal booking date will be taken as a proxy for FNP enrolment date (index date). Controls will be women that would have been potentially eligible for FNP, but whose antenatal booking occurred during a period when local FNP teams were not enrolling women. Alongside the eligibility criteria, using the start and end dates of FNP enrolment (including any intervals when cohorts ceased enrolment) provided to us by the FNP SIS, eDRIS will identify individual Controls with antenatal booking dates:

i. in the 12 months prior to initiation of FNP enrolment (Pre-FNP);

ii. in the 12 months post FNP enrolment (Post-FNP);

iii. between periods of FNP enrolment (i.e. when enrolment was temporarily suspended due to caseload capacity, usually up to a maximum concurrent caseload of 25 women, being reached) (Interval).

Further ineligibility criteria are applied to women during the FNP enrolment process, such as planning to place the child for adoption or moving out of the area, but cannot be applied in the selection of Controls as they are not assessed in the SMR02 (or any other dataset) at date of booking.

Within the enrolment period, a second cohort of women exist consisting of women eligible for enrolment into the programme during a period of active FNP enrolment and who:

iv. were approached for FNP but not enrolled to the programme;

v. were not approached (e.g. insufficient capacity in team to offer to all eligible women; near end of enrolment period and caseloads nearly full).

Following advice from the Public Benefit and Privacy Panel (PBPP), it was not possible to access individual level data for these women (in iv and v). However, it is important to note that these groups may have different demographic, social or personal characteristics from those that enrolled in the programme and may differ non-randomly from the Cases for some variables. As it would be beneficial to the evaluation to understand the characteristics of (iv) and (v) above and understand if they differ from Cases and Controls in anyway, aggregate statistics will be provided by eDRIS to the study team. 

### Identifying children

A mother-child link is available within the SMR02 enabling a flag for children born after the FNP enrolment date (Cases) / antenatal booking date (Controls) to be derived. 

### Data Controllers and Data Processors

#### NHS National Services Scotland (NSS)

NSS is the Data Controller for centrally held health data on Scottish patients made available following approval from the PBPP. Datasets include Outpatient Attendance, Acute Inpatient and Day Case, Maternity Inpatient and Mental Health, Child Health, Birth Record and Accident & Emergency [26]. SMR 02 contains the maternity inpatient and day case data for all women registered to receive maternity health care.

#### Electronic Data Research and Innovation Service (eDRIS)

eDRIS provides a single point of contact to assist researchers in study design, approvals and data access for projects using routinely collected health data in Scotland [27]. The role of eDRIS for this study is to advise on the application submitted to the PBPP, liaise with the trusted third party indexing team (National Record for Scotland, NRS) and the various data controllers on behalf of the study. eDRIS will also oversee all data transfer and linkage to create the study cohort. eDRIS are data processors for this study. The PBPP is a governance structure of NHS Scotland with a remit to carry out information governance scrutiny of requests for linkage and/or access to individual level health data on behalf of NHS Scotland [28].

#### Health Boards (HB)

Each HB in Scotland acts as the Data Controller for the data held on the FNP Scottish Information System (FNP SIS), a national database of FNP data accessible to Family Nurse (FN) teams. Agreement has been sought to allow these data to be processed by eDRIS for this study. Data include both identifiable data that will enable linkage to FNP Clients’ routine data from health, education and social care as well as clinical data recorded by FNs during their visits.

#### Education Analytical Service (EAS) Division

The EAS is a division of the Scottish Government who are data controllers for Education and Social Care data. Requests for data are made through the Data Access Panel. The panel assesses the objectives and data protection implications in relation to each request as well as the security arrangements [29]. 

#### National Records for Scotland (NRS)

The NRS indexing team ensure robust linkage of education data to the research population spine. The pupil census has been matched to the spine to create an anonymised “read-through” index key which NRS and EAS hold at an individual-level.

#### National Services Scotland Safe Haven

**Figure 1: Model of pseudonymised data linkage: FNP Scotland Research Database:**
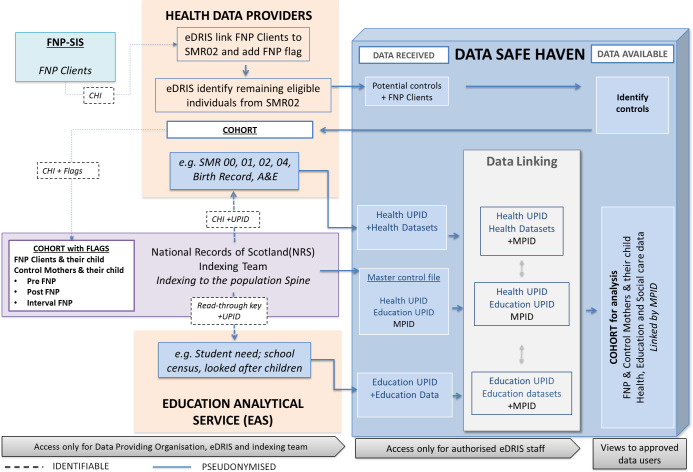
National Services Scotland Safe Haven is a secure environment in which data are linked and stored. In this case, FNP Client data (Cases) from the Family Nurse Partnership Scottish Information System (FNP SIS) is sent to eDRIS to be linked to health datasets to identify Controls and obtain health outcome data. The cohort is linked to Education Analytics Service (EAS) data using the population spine to enable linkage to education and social care data. Approved access is provided either remotely or via a secure access point and set up by eDRIS. Both access methods allow trusted and authorised researchers to analyse individual level data while maintaining confidentiality.

NSS is a secure environment in which data are linked and stored. Approved access is provided either remotely or via a secure access point and set up by eDRIS. Both access methods allow trusted and authorised researchers to analyse individual level data while maintaining confidentiality. Remote access to the safe haven is via Citrix software using an accredited organisation’s PC / laptop. The data linkage process is shown in Figure 1.

FNP SIS – Family Nurse Partnership Scottish Information System;

SMR – Scottish Morbidity Record

CHI – Community Health Index

UPID – Unique Person ID

MPID – Master Person ID.

### Data sources and outcomes

Table 2 provides a summary of the datasets requested for use in this study alongside the Data Controller and the relevant panel to approve data access.

**Table 2: Maternal baseline characteristics measured at (or before) date of antenatal booking/enrolment:** PBPP: Public Benefit and Privacy Panel; EAS: Education Analytical Services; NHS NSS: NHS National Services Scotland; SG: Scottish Government; A&E: Accident and Emergency; CHSP: Child Health Systems Programme; HB: Health Board; NRS: National Record for Scotland; CHI: Community Health Index; SMR: Scottish Morbidity Record.

Source datasets	Brief overview of dataset	Data Controller	PBPP	EAS
SMR 00 - Outpatient Attendance	Comprises data from 1997 for patients on new and follow up appointments at outpatient clinics in all specialities (except A&E and Genito urinary Medicine).	NHS NSS	✓
SMR 01 - General/Acute Inpatients and Day Case	Comprises episode level data from 1981 on hospital inpatient and day case charges from acute specialities.	NHS NSS	✓
SMR 02 - Maternity Inpatients and Day Case	Comprises episode level data from 1975 every time a mother goes in for an obstetric event and includes information on mother and baby characteristics, birth weight, gestational age, mode of delivery, induction and outcome of pregnancy and where a baby is delivered.	NHS NSS	✓
SMR 04 - Mental Health Inpatient and Day Case	Comprises episode level data since 1981 on patients that are receiving care at psychiatric hospitals at the point of both admission and discharge.	NHS NSS	✓
Community prescribing and dispensing	Contains all NHS prescriptions dispensed in the community in Scotland	NHS NSS	✓
NRS: Death data	Contains details of every death in Scotland	NHS NSS	✓
CHI: Demographics	Every person registered with a GP in Scotland is allocated a 10-digit CHI number from a centrally maintained register. The register contains data on address, postcode, GP, date of birth, region of registration and, where relevant, date of death.	NHS NSS	✓
Unscheduled Care: Accident and Emergency (A&E)	Contains data on patient attendances at Emergency Departments, Minor Injuries Units and community hospital A&Es across NHS Scotland.	NHS NSS	✓
CHSP-Pre-School- Health Visitor first visit (~10 days); 6-8 week review; 27-30 month review; unscheduled review	Contains data on the findings and outcome of the child health reviews for all children in Scotland. Child health reviews incorporate assessment of children's health, development, and wider wellbeing alongside provision of health promotion advice and parenting support.	NHS NSS	✓
CHSP-School - Primary 1 - screening and assessment	A Primary 1 review is offered to all children in Scotland in mainstream and special state schools at the age of 5 as part of the wider child health programme. It contains information on include height and weight measurements, and recording of diagnoses/concerns. Following the primary 1 screening some children have a further assessment which reviews the child's development (e.g. gross motor, fine motor, speech and language, social skills and behaviour).	NHS NSS	✓
FNP Scottish Information System	Contains information on the FNP programme delivery from enrolment in the programme.	Local HBs	✓
School/Pupil Census	Contains information on early learning and childcare provision, pupils, teachers, and school buildings collected from all Local Authority and Grant-aided schools and school centres on publicly funded schools.	SG		✓
Attendance, Absence and Exclusions	Contains information from schools on pupils attendance and absence from schools and the reasons for this. It also includes exclusions.	SG		✓
School Leavers (Summer and Christmas)	Contains information on pupils in each school year who leave school having attained the minimum school leaving age.	SG		✓
Skills Development Scotland: destinations	Contains data on 16-19 year olds in Scotland on school leaver destinations and type of destination (higher education, further education, employment, training, unemployed).	SG		✓
Children and Young People: Looked after children	Each Scottish Local Authority is asked to submit a return providing information on the Looked After Children for which they are responsible.	SG		✓
Children and Young People: Child protection register	Child protection data via the from Local Authorities’ Management Information Systems. The information collected here is the primary national data source on child protection in Scotland.	SG		✓
Scottish Credit and Qualifications Framework	Contains information on the qualifications awarded to learners in Scotland.	SG		✓
Achievement of Curriculum for Excellence levels collections	Achievement of Curriculum for Excellence for all pupils in the stages of Primary (P)1, P4, P7, and Secondary (S)3, who are based on the roll of the school, and for all pupils based in standalone school/units. Each of the following will be collected: Numeracy, Reading, Writing and Listening and Talking.	SG		✓

The specification for this evaluation of FNP requires there be no pre-specified primary outcome(s). The outcomes of this study closely follow the key activities and outcomes in the FNP programme’s logic model [24], covering maternal health, child health and development, and maternal life course and are listed in Table 3.

The ability to report on outcomes will depend on several factors such as geographical and study cohort coverage, data quality (completeness and bias). As a result outcomes have been categorised into either short- or medium-term or descriptive. Short term outcomes (n=25) map well to the logic model, are known to have good data quality, and coverage across Scotland and our study cohort (thus maximising the number that can be formally analysed using statistical methods). These outcomes are likely to be associated with the pregnancy and birth period, and up to the child’s second birthday (e.g. child health outcomes). Medium-term outcomes (n=9) rely on data only measured in the period after the second birthday (e.g. school-based outcomes at age four-years of age onwards) and the population included in the analysis would be restricted as a result. These short- and medium-term outcomes will be formally analysed using statistical modelling and will compare between participants who did (Cases) and did not receive FNP provision (Controls).

Descriptive outcomes (n=20) are those where the direction of the FNP effect is either uncertain, outcomes are rare, or where the data are classed as experimental statistics (i.e. a type of official statistic that is undergoing development such as child attainment).

## Analysis

### Power calculation

In this retrospective study, the sample size is fixed. The Evaluability Assessment estimated around 3000 births in FNP cohorts between 2010 and 2015 and around 6000 in the Controls [24]. This large sample size would permit very precise estimation of overall intervention effects for a primary or co-primary outcomes. However as specified by Scottish Government, there is no pre-specified prioritisation of the short- or medium-term outcomes and as such no power calculation is necessary. 

### Identification of Cases and Controls

#### Cases

The number of Cases identified and received from FNP SIS and matched to the SMR02 dataset by eDRIS will be reported. Additional checks will be made on the eligibility of FNP clients using SMR02 fields (age at enrolment vs age at booking, age at Last Menstrual Period gestational age at enrolment vs. booking). This will allow us to measure the robustness of these fields used to identify the Controls.

#### Controls

In a natural experiment, to enable an unbiased comparison of Cases and Controls, measured risk factors associated with outcomes (known as covariates) should be sufficiently similar (and thus balanced) for both exposure groups. As the potential Controls would have been eligible for enrolment on FNP during a period of non-recruitment, they are already a more homogenous comparison population (according to age, area and parity). It is likely, therefore, that the Controls will be sufficiently similar to Cases. However, a possible threat to an unbiased comparison is the enrolment of mothers into FNP on criteria other than age, area and parity, and balance may not be adequate. For example, if mothers who are approached but not enrolled in FNP differ from those who are enrolled for characteristics that are associated with variation in outcomes, comparisons with all eligible Controls may under- or overestimate any effect of FNP. One way to address this is to match the population further as it may provide a more valid estimate of effects because only women with similar observed characteristics are included. The disadvantages of this approach would be that not all Cases would be matched to Controls, which would risk the exclusion of Cases and reduce the sample size. The approach taken here will retain all Controls and given the possibility of a homogeneous comparison group, results will be more generalizable, and result in higher power.

### Descriptive analysis: Cases and Controls

Measurable pre-recruitment/at booking maternal demographics and socioeconomic covariates associated with the FNP enrolment and outcomes were decided a priori (Table 4). Covariates should be those that are not affected by exposure and measured before recruitment into FNP. The maternal characteristics will be described in Cases and all Controls using summary statistics (e.g. N (%), mean (standard deviation (SD))).

**Table 3: Maternal baseline characteristics measured at (or before) date of antenatal booking/enrolment:** SMR: Scottish Morbidity Record; FNP SIS – Family Nurse Partnership Scottish Information System;

Dataset	Variable
SMR02	Health Board based on postcode at antenatal booking date
SMR02	Scottish Index of Multiple Deprivation (SIMD) quintile
SMR02	Ethnic Group (White/Other)
SMR02	Age at antenatal booking (years)
SMR02/FNP SIS	Age at last menstrual period (LMP) (years)
SMR02/FNP SIS	Completed weeks of gestation at antenatal booking/FNP enrolment date
SMR02	Maternal Height/Weight/Body Mass Index at booking date
SMR02	Booking Smoking History (never/non-smoker/current/former)
SMR02	Smoker during pregnancy recorded at booking (never/non-smoker/former/current)
SMR02	Drug misuse during pregnancy recorded at booking (yes/no)
SMR02	Illegal drugs/inappropriate injection of prescribed drugs at booking (yes/no)
SMR02	Typical weekly alcohol consumption at booking (units)
SMR02	Diabetes (pre-existing, gestational, yes but time of diagnosis unknown/no diabetes during this pregnancy, not known)
Dispensing	Drugs ever dispensed for asthma (yes/no)
Dispensing	Drugs ever dispensed for mental ill health (yes/no)
SMR02	Previous pregnancy (yes/no)
SMR02	Outcome of pregnancy (live/stillbirth/termination)
Child protection register	Ever been on child protection register
Looked after children	Ever looked after child
School/Pupil Census	Ever had a Free School Meal
School/Pupil Census	Ever had a student need
Attendances, Absences and Exclusions	Ever been excluded
Attendances, Absences and Exclusions	Attendance rate
Mother left school or still in school at booking date	School leavers (Summer and Christmas)

### Main analyses

With no primary outcome, equal importance will be given to each short and medium term outcome. All comparative analyses will therefore be exploratory, pre-specified and conducted on an intention to treat (ITT) basis. ITT in this study means that the analysis will include everyone who started the programme, according to their original ‘allocation’, i.e. the intervention group will be women enrolled in FNP regardless of the treatment (intervention) they actually received.

All analyses will compare outcomes (intervention effect) between the two groups (Cases and Controls) using multilevel regression models, to allow for clustering of outcome within NHS HB, and FNP team/cohort (where more than one team runs within a HB). Multiple births will be analysed as separate children and the potential clustering of outcome will not be taken account for in analyses due to the small numbers expected. Intervention effects will also be examined over time and between different geographical areas (HB and team/cohort) by fitting multilevel models and interactions (group x year). Alongside the estimate of effect, for all outcomes a 95% confidence interval (CI) and unadjusted p-value will be presented.

Binary outcomes will be modelled using a logistic model and presented as odds ratios comparing the odds of an event in a case compared with the Control. For continuous outcomes a multilevel linear model will be fitted and results presented as a difference in means (Case minus Control group). Time to event analyses (e.g. cessation of breastfeeding, time to subsequent birth) will be analysed using a proportional hazards regression model and results presented as hazard ratios. We will ascertain if the proportional hazards assumption has not been violated by inspecting the log (-log(survival)) plot and Schoenfeld residuals. Count data will be analysed using a Poisson multilevel model. If the distribution of events display signs of over dispersion (greater variance than might be expected in a Poisson distribution), then a Negative Binomial model will be used. Results will be presented as the incidence rate ratio in the case arm compared to the Control group. The impact of FNP visits (dosage of intervention) on outcomes will be explored as a sensitivity analysis. Adherence will be defined as the number of FNP visits that a Client received during their programme enrolment overall or by phase (pregnancy, infancy, toddler), dependant on outcome. A number of other sensitivity analyses are proposed including adjustment for any imbalance in confounders (pre-exposure maternal and baby characteristics) (note that these will be assessed for the differing denominators (study populations) dependent on outcome) and adjustment for multiple testing.

#### Subgroups

We will examine the effect of FNP on pre-specified outcomes by modelling interactions between FNP uptake and pre-specified maternal baseline characteristics such as ever been on the child protection register/ looked after child, substance misuse issues and child demographics such as gender. These analyses are not powered for and will be exploratory in nature. Effect sizes alongside 95% confidence intervals and p-values will be reported.

A detailed statistical analysis plan will be written prior to access to data and signed off by the co-lead for the project. The reporting and presentation of results will be in accordance with the GUILD, STROBE, RECORD and TREND guidelines to ensure the comprehensive reporting of this observational non-randomized evaluation of a public health intervention [31–34]. SPSS and Stata will be used for all analyses [35, 36]. We will adhere to the NSS Statistical Disclosure Control protocol [37, 38]. 

### Study timelines

The study started in July 2016, data will be made available for analysis to begin in January 2020 and will report to the Scottish Government in 2020. 

### Patient and Public Involvement

A lay representative on the study steering committee has provided input on the public facing material describing the study (i.e. lay summary). The PBPP have a number of public representatives contributing to the Tier 2 application review and decision-making.

## Discussion

The study protocol is based upon the specification of FNP commissioned by the Scottish Government. This study design is novel for the evaluation of the FNP/NFP programmes which are primarily evaluated with an RCT. However, assessment of effectiveness in evaluation is limited to outcomes available from routinely collected data. The outcomes for this study have been selected by matching routinely collected administrative data to the Scottish FNP logic model, which is based on the underlying programme theory. Therefore, they have been selected on the basis that they are outcomes FNP aims to influence and there is routine data available to assess the outcome. The Controls are well defined and can be identified from routine data sources. However, one limitation is that women who were approached but not enrolled to the programme were removed from the Cases, but those who might have been approached and subsequently not enrolled will still be present in the control group. Since access to these individuals’ data was not permitted, women of similar characteristics in the Controls cannot be identified to remove them. We can however describe these women who did not take up the programme in a period of recruitment (either not approached or not enrolled) and assess if they are different in any way to the case and control groups. If they are not different on the measured factors, then they do not pose a problem. If they are then the potential bias it might bring to the evaluation will be reflected on in the discussion of the main paper.

There are no primary outcomes selected for this evaluation. The implication for this approach is that the large number of statistical tests (34 short- and medium-term proposed in this study) performed will mean that there is an increased risk of finding a significant result by chance (false-positive error) i.e. given a 0.05 alpha there is an 82% (1-0.95^34^) chance that at least one of these tests are statistically significant by chance when the conclusion is not true in the population. Recently, James Heckman and colleagues re-assessed the findings of the Memphis trial of NFP using a ‘step-down’ approach to address multiple significance testing [39]. They concluded that fewer treatment effects survive this more conservative approach but note strong effects surviving, especially for longer term effects amongst boys. For individual studies, other correction methods such as Bonferroni have been suggested but are criticised as being overly conservative. On the other hand, if the sample size is sufficiently large and a moderate number of tests are being carried out, the Bonferroni correction will make little difference to the magnitude of effect sizes that can be detected [40]. The recommendations given by the authors are to report the actual p-values without a correction, and then indicate the number of tests and what the Bonferroni correction threshold would be, allowing the reader to assess the evidence themselves. The more recent trials of FNP including our own (BB:0-2) have taken the standard approach of identifying primary outcomes (in the case of BB:0-2, two primary outcomes per population were assessed) and adjusting the alpha accordingly. In this commissioned service evaluation the proposed analysis are exploratory and results should be interpreted with caution.

There are various governance and contractual requirements placed on this study, many of which were identified and required by the PBPP and/or EAS panel prior to final approval. All researchers have completed the information governance training required by eDRIS to evidence their “approved researcher” status. Thirty-three data sharing and/or data processing agreements were set up between HBs, NSS, NRS, EAS and SG to allow the transfer, processing and storage of data for this study. Setting up the agreements took time and posed a risk to the delivery of the project if HBs didn’t support the work however support was provided by the Scottish Government to facilitate this process. 

## Conclusions

This is an anonymised data linkage study evaluating the FNP programme in Scotland. It will provide the Scottish Government with indications of the potential effectiveness of this programme in Scotland to contribute to their policy making decisions on early years interventions.

## Acknowledgments

We gratefully acknowledge the Scottish Government who funded the service evaluation of FNP upon which this present work has been drawn. The Centre for Trials Research receives funding from Health and Care Research Wales and Cancer Research UK. The work was undertaken with the support of The Centre for the Development and Evaluation of Complex Interventions for Public Health Improvement (DECIPHer), a UK Clinical Research Collaboration (UKCRC) Public Health Research Centre of Excellence. Joint funding (MR/KO232331/1) from the British Heart Foundation, Cancer Research UK, Economic and Social Research Council, Medical Research Council, the Welsh Government and the Wellcome Trust, under the auspices of the UKCRC, is gratefully acknowledged. We acknowledge the Family Nurse Partnership Evaluation and Research Advisory Group (ERAG) who informed the identification of potential outcomes for this evaluation. We acknowledge the study steering committee’s role in supporting the study: John Frank (Committee Chair); Sandra Baldwin; Pat Hoddinott; David Low and Gordon Taylor.

## Declarations

### Ethics approval and consent to participate

South East Scotland Research Ethics Service confirmed the study will not require an NHS Ethical Review as it is a service evaluation.

### Consent for publication

Not Applicable.

### Availability of data and material

Not Applicable.

### Funding statement

This project was funded by the Scottish Government Children and Families Directorate [project reference CASE/290185]. The manuscript was prepared by the authors and in consultation with the Scottish Government to represent the contracted service evaluation of FNP in Scotland.

### Authors’ contributions

RCJ and MR are co-chief investigators of the study. All authors have contributed to and are responsible for the final design of the study. FLW is responsible for study management. RCJ and ML are responsible for statistical planning, data management and data analysis. All authors read and approved the final manuscript.

## Abbreviations

A&E	Accident and Emergency
AGA	Appropriate for gestational age
BB:0-2	Building Blocks Trial
CHI	Community Health Index
CHP	Community Health Partnership
CHSP	Child Health Systems Programme
CI	Confidence Intervals
EAS	Education Analytical Service
eDRIS	Electronic Data Research and Innovation Service
FN	Family Nurse
FNP	Family Nurse Partnership
HB	Health Board
IPTW	Inverse Probability of Treatment Weighting
LMP	Last Menstrual Period
MRC	Medical Research Council
NFP	Nurse Family Partnership
NRS	National Record for Scotland
NSS	NHS National Services Scotland
PBPP	Public Benefit & Privacy Panel
PP	Post-Partum
RCT	Randomised Controlled Trial
SIS	Scottish Information System
SMR	Scottish Morbidity Record

## References

[1] Chen X-K, Wen SW, Fleming N, Demissie K, Rhoads GG, Walker M. Teenage pregnancy and adverse birth outcomes: a large population based retrospective cohort study. Int J Epidemiol. 2007;36(2):368-373. 10.1093/ije/dyl284 17213208

[2] McAndrew F, Thompson J, Fellows L, Large A, Speed M, Renfrew MJ. Infant feeding survey 2010. Leeds; 2012.

[3] Manktelow BN, Smith L, Prunet C, Smith P, Boby T, Hyman-Taylor P, et al MBRRACE-UK Perinatal Mortality Surveillance Report, UK Perinatal Deaths for Births from January to December 2015. Leicester; 2017.

[4] Ekeus C, Christensson K, Hjern A. Unintentional and violent injuries among pre-school children of teenage mothers in Sweden: a national cohort study. J Epidemiol Community Heal. ;():–52004;58(8):680-685. 10.1136/jech.2003.015255 PMC1732862 15252071

[5] Graham H, Inskip HM, Francis B, Harman J. Pathways of disadvantage and smoking careers: evidence and policy implications. J Epidemiol Community Health. 2006;60 Suppl 2(suppl 2):7-12 [accessed 2017 Nov 27]. Available from: http://www.ncbi.nlm.nih.gov/pubmed/17708005 10.1136/jech.2005.045583 PMC2491894 17708005

[6] U.S. Department of Health & Human Services. Home Visiting Evidence of Effectiveness. [accessed 2017 Nov 27]. Available from: https://homvee.acf.hhs.gov/

[7] Olds D. Prenatal and Infancy Home Visiting by Nurses: From Randomized Trials to Community Replication. Prev Scien. 2002;3(3):153–72. 10.1023/a:1019990432161 12387552

[8] Bronfenbrenner U. Toward an Experimental Ecology of Human Development. Am Psychol. 1977;32(7):513 [accessed 2017 Nov 27]. Available from: https://pdfs.semanticscholar.org/a857/783a2bfc8aef8c93c200b3c635549237b434.pdf

[9] Bandura A. Self-efficacy: Toward a Unifying Theory of Behavioral Change. Psychol Rev. 1977;84(2):191-215 [accessed 2017 Nov 27]. Available from: https://pdfs.semanticscholar.org/e8af/4369e0533210860587b7add0c566b74b963a.pdf 847061 10.1037//0033-295x.84.2.191

[10] Bowlby E. Loss-Sadness and Depression: Attachment and Loss. Random House; 2008.

[11] Olds D, Henderson CR, Cole R, Eckenrode J, Kitzman H, Luckey D, et al Long-term Effects of Nurse Home Visitation on Children’s Criminal and Antisocial Behavior 15-Year Follow-up of a Randomized Controlled Trial. JAMA. 1998;280:1238-1244. 10.1001/jama.280.14.1238 9786373

[12] Olds D, Henderson CR, Kitzman H, Cole R. Effects of Prenatal and Infancy Nurse Home Visitation on Surveillance of Child Maltreatment. Pediatrics. 1995;95(3) [accessed 2017 May 5]. Available from: http://pediatrics.aappublications.org/content/95/3/365.short 7862474

[13] Olds DL, Henderson CR, Kitzman H. Does Prenatal and Infancy Nurse Home Visitation Have Enduring Effects on Qualities of Parental Caregiving and Child Health at 25 to 50 Months of Life? Pediatrics. 1994;93. 8265329

[14] Olds DL, Henderson CR, Chamberlin R, Tatelbaum R. Preventing Child Abuse and Neglect: A Randomized Trial of Nurse Home Visitation. Pediatrics. 1986;78(1) [accessed 2017 May 5]. Available from: http://pediatrics.aappublications.org/content/pediatrics/78/1/65.full.pdf 2425334

[15] Dennis Luckey HW, Henderson CR, Hanks C, Bondy J, David Olds JL, Kitzman H, et al Development: Age 6 Follow-Up Results of a Randomized Trial Effects of Nurse Home-Visiting on Maternal Life Course and Child Effects of Nurse Home-Visiting on Maternal Life Course and Child Development: Age 6 Follow-Up Results of a Randomized Trial. 2004; Available from: http://www.pediatrics.org/cgi/content/full/114/6/1550 10.1542/peds.2004-0962 15574614

[16] Olds DL, Robinson J, Pettitt L, Luckey DW, Holmberg J, Ng RK, et al Effects of Home Visits by Paraprofessionals and by Nurses: Age 4 Follow-Up Results of a Randomized Trial. Pediatrics. 2004;114(6):1560-1568. 10.1542/peds.2004-0961 15574615

[17] Olds DL, Henderson CR, Tatelbaum R, Chamberlin R. Improving the Life-Course Development of Socially Disadvantaged Mothers: A Randomized Trial of Nurse Home Visitation. Am J Public Health. 1988;78(11):1436-1445 [accessed 2017 Nov 27]. Available from: http://ajph.aphapublications.org/doi/pdf/10.2105/AJPH.78.11.1436 3052116 10.2105/ajph.78.11.1436 PMC1350235

[18] Olds DL, Kitzman H, Hanks C, Cole R, Anson E, Sidora-Arcoleo K, et al Effects of Nurse Home Visiting on Maternal and Child Functioning: Age-9 Follow-up of a Randomized Trial. Pediatrics. 2007;120(4):e832–45. 10.1542/peds.2006-2111 PMC2839449 17908740

[19] Robling M, Bekkers MJ, Bell K, Butler CC, Cannings-John R, Channon S, et al Effectiveness of a nurse-led intensive home-visitation programme for first-time teenage mothers (Building Blocks): A pragmatic randomised controlled trial. Lancet. 2016;387:146-155. Available from: 10.1016/S0140-6736(15)00392-X 26474809 PMC4707160

[20] Owen-Jones E, Bekkers M-J, Butler CC, Cannings-John R, Channon S, Hood K, et al The effectiveness and cost-effectiveness of the Family Nurse Partnership home visiting programme for first time teenage mothers in England: a protocol for the Building Blocks randomised controlled trial. BMC Pediatr. 2013;13:1 10.1186/1471-2431-13-114 23919573 PMC3750355

[21] Mejdoubi J, Van den Heijkant SCCM, Van Leerdam FJM, Crone M, Crijnen A, HiraSing RA. Effects of nurse home visitation on cigarette smoking, pregnancy outcomes and breastfeeding: A randomized controlled trial. Midwifery. 2014;30(6):688-695. 10.1016/j.midw.2013.08.006 24041564

[22] Mejdoubi J, Van Den Heijkant SCCM, Van Leerdam FJM, Heymans MW, Crijnen A, Hirasing RA. The effect of VoorZorg, the dutch nurse-family partnership, on child maltreatment and development: A randomized controlled trial. PLoS One. 2015;10(4). 10.1371/journal.pone.0120182 PMC4382107 25830242

[23] Ormston R, McConville S, Gordon J. Evaluation of the Family Nurse Partnership Programme in NHS Lothian, Scotland: Summary of key learning and implications. 2017 [accessed 2017 Nov 27]. Available from: http://www.gov.scot/resource/0044/00444851.pdf

[24] Wimbush E, Geddes R, Woodman K, Craig P, Jepson R. Evaluability assessment of the Family Nurse Partnership in Scotland. 2015;(March). Available from: http://www.healthscotland.com/uploads/documents/26102-Family%20Nurse%20Partnership%20Evaluability%20Assessment%20Report.pdf

[25] Craig P, Cooper C, Gunnell D, Haw S, Lawson K, Macintyre S, et al Using natural experiments to evaluate population health interventions: new Medical Research Council guidance. J Epidemiol Community Heal. 2012;66:1182-1186 [accessed 2017 Jul 19]. Available from: http://jech.bmj.com/content/jech/66/12/1182.full.pdf doi: 10.1136/jech-2011-200375 PMC3796763 22577181

[26] ISD Scotland. SMR Datasets. [accessed 2017 Nov 27]. Available from: http://www.ndc.scot.nhs.uk/Data-Dictionary/SMR-Datasets/

[27] ISD Scotland. Electronic Data Research and Innovation Service (eDRIS). [accessed 2017 Nov 27]. Available from: http://www.isdscotland.org/Products-and-services/Edris/FAQ-eDRIS/index.asp

[28] Public Benefit and Privacy Panel for Health and Social Care. [accessed 2017 Nov 27]. Available from: http://www.informationgovernance.scot.nhs.uk/pbpphsc/

[29] Scottish Government. School Education - Data Access. [accessed 2017 Nov 27]. Available from: http://www.gov.scot/Topics/Statistics/Browse/School-Education/DataAccess

[30] Gardosi J. GROW documentation. 2017 [accessed 2017 Nov 27]. Available from: https://www.gestation.net/grow_documentation.pdf

[31] Hemkens LG, Benchimol EI, Langan SM, Briel M, Kasenda B, Januel J-M, et al The reporting of studies using routinely collected health data was often insufficient. J Clin Epidemiol. 2016;79:104-11 [accessed 2018 Nov 27]. Available from: 10.1016/j.jclinepi.2016.06.005 27343981 PMC5152936

[32] Benchimol EI, Smeeth L, Guttmann A, Harron K, Moher D, Petersen I, et al The REporting of studies Conducted using Observational Routinely-collected health Data (RECORD) Statement. PLoS Med. 2015;12(10):e1001885 [accessed 2017 Sep 8]. Available from: . 10.1371/journal.pmed.100188533 26440803 PMC4595218

[33] Des Jarlais DC, Lyles C, Crepaz N, the TREND Group. Improving the reporting quality of nonrandomized evaluations of behavioral and public health interventions: the TREND statement. Am J Public Health. ;():–62004;94(3):361-366 [accessed 2017 Nov 27]. Available from: 10.2105/ajph.94.3.361 14998794 PMC1448256

[34] Gilbert R, Lafferty R, Hagger-Johnson G, Harron K, Zhang L-C, Smith P, et al GUILD: GUidance for Information about Linking Data sets†. J Public Health (Bangkok). 2017;(November):1-8. Available from: 10.1093/pubmed/fdx037 PMC5896589 28369581

[35] IBM. IBM SPSS Statistics for Windows: Version 22.0. IBM Corp; 2013.

[36] StataCorp. Stata Statistical Software: Release 15. 2017. 2017.

[37] NHS NSS. Statistical Disclosure Control Protocol. 2015.

[38] eDRIS Team. Researcher guide: requesting outputs from Safe Haven and disclosure control. 2018.

[39] Heckman JJ, Holland ML, Makino KK, Pinto R, Rosales-Rueda M. An Analysis of the Memphis Nurse-Family Partnership Program. NBER Work Pap. 2017;23610 Available from: http://www.nber.org/papers/w23610

[40] VanderWeele TJ, Mathur MB. Some Desirable Properties of the Bonferroni Correction: Is the Bonferroni correction really so bad? Am J Epidemiol. 2019;188(3):617-618. 10.1093/aje/kwy250 30452538 PMC6395159

